# An Intriguing Case of Multisystem Inflammatory Syndrome in an Adult Patient with Remote Infection with COVID-19 and Acute *Chlamydia*

**DOI:** 10.1155/2021/6620240

**Published:** 2021-09-29

**Authors:** Madhuri Chandnani, William A. Charini, Anil Jha, Chetan Dodhia, Eduardo Haddad

**Affiliations:** ^1^Department of Hospital Medicine/Internal Medicine, Lawrence General Hospital, Lawrence, MA, USA; ^2^Department of Infectious Diseases, Lawrence General Hospital, Lawrence, MA, USA; ^3^President of Medical Staff and Department of Nephrology, Lawrence General Hospital, Lawrence, MA, USA

## Abstract

**Introduction:**

COVID-19 is associated with a broad range of immune inflammatory phenomena, with different manifestations in adults and children. We describe a case of COVID-19-related multisystem inflammatory syndrome in an adult (MIS-A), similar to that described in children (MIS-C), which may have been set off by an unrelated secondary infection.

**Case:**

A 27-year-old male patient presented with acute epididymitis secondary to acute *Chlamydia* infection that progressed to multisystem inflammatory failure with respiratory failure requiring endotracheal intubation and mechanical ventilation, cardiogenic shock with heart failure, and gastrointestinal and renal dysfunction. He tested negative for severe acute respiratory syndrome coronavirus 2 (SARS-CoV-2) by reverse transcriptase-polymerase chain reaction on a nasopharyngeal swab thrice within 4 days of presentation, but positive for SARS-CoV-2 immunoglobulin G antibody signifying remote infection. The patient was treated with tocilizumab and steroids, along with doxycycline for concurrent *Chlamydia* infection, resulting in dramatic improvement in all organ function. We suspect that *C. trachomatis* infection in this instance may have triggered an aberrant immune response that was shaped by prior exposure to SARS-CoV-2.

**Conclusion:**

We present a case of an adult patient with acute *Chlamydia trachomatis* infection occurring in the wake of asymptomatic (or at least unrecognized) COVID-19 resulting in MIS-A. Clinicians should be alert to the possibility of other such unusual reactions occurring in the aftermath of COVID-19. This case also highlights the importance for clinicians who care for adult patients of being familiar with the multisystem inflammatory syndrome of children, as an identical syndrome may occur in adult patients.

## 1. Introduction

As of August 6, 2021, severe acute respiratory syndrome coronavirus 2 (SARS-CoV-2) causing coronavirus disease 2019 (COVID-19) has led to 200,840,180 cases and 4,265,903 deaths worldwide [1] and 35,392,284 cases and 612,958 deaths in the United States [2]. At the beginning of the COVID-19 pandemic, a high incidence of severe acute respiratory distress syndrome was reported, mainly in adults. The disease in children was thought to be mild. Then, reports appeared in United Kingdom [3], Italy [4], and United States [5, 6] of a high rate of critical illness in children affected with COVID-19 exhibiting inflammatory multisystem failure.

The United States Center for Disease Control (CDC) formally named this entity multisystem inflammatory syndrome in children (MIS-C) associated with COVID-19 and defined a confirmed case of MIS-C as follows:

1. An individual aged <21 years presenting with fever, laboratory evidence of inflammation, and evidence of clinically severe illness requiring hospitalization, with multisystem (≥2) organ involvement (cardiac, renal, respiratory, hematologic, gastrointestinal, dermatologic, or neurological)
2. No alternative plausible diagnoses
3. Positive for current or recent SARS-CoV-2 infection by RT-PCR (reverse transcriptase-polymerase chain reaction), serology, or antigen test, or COVID-19 exposure within the 4 weeks prior to the onset of symptoms [7]

Then, in October 2020, CDC published a case series of multisystem inflammatory syndrome in adults (MIS-A) associated with SARS-CoV-2 [8], in which the authors established a working case definition of MIS-A as follows:

1. A severe illness requiring hospitalization in a person aged ≥21 years
2. A positive test result for current or previous SARS-CoV-2 infection (nucleic acid, antigen, or antibody) during admission or in the previous 12 weeks
3. Severe dysfunction of one or more extrapulmonary organ systems (e.g., hypotension or shock, cardiac dysfunction, arterial or venous thrombosis or thromboembolism, or acute liver injury)
4. Laboratory evidence of severe inflammation (e.g., elevated CRP, ferritin, D-dimer, or interleukin-6)
5. Absence of severe respiratory illness (to exclude patients in whom inflammation and organ dysfunction might be attributable simply to tissue hypoxia)

Regarding criterion (5), the authors pointed out that the working case definition also excluded patients with severe respiratory dysfunction in order to distinguish MIS-A from severe COVID-19 itself.

It is becoming increasingly clear that COVID-19 is associated with a broad range of immune inflammatory phenomena, with different manifestations in adults and children. The pathophysiology of MIS remains unclear. Some of these manifestations occur in the setting of acute SARS-CoV-2 infection, and some of them are postinfection phenomena. We describe a case in point of full-blown COVID-19-related MIS-A associated with respiratory dysfunction, probably set off by an unrelated secondary infection.

## 2. Case

A 27-year-old male of Afro-Latino ethnicity with no significant past medical history presented in May 2020 to a community hospital emergency room (ER) with complaints of a dull, lower abdominal pain associated with subjective fever and loss of appetite over 3 days. Review of systems was notable for dysuria and penile discharge, which he self-treated with 3 doses of ampicillin 1 g and which had resolved by the time of presentation.

His vitals in the ER included a temperature of 101.5 F, a pulse of 68/min, a blood pressure (BP) of 114/74 mmHg, a respiratory rate of 20/min, and an oxygen saturation of 97% on room air (RA). He was noted to have tenderness in the inguinal and hypogastric regions bilaterally, but no penile discharge was evident. Blood work was remarkable for mild leukocytosis of 11.3 K/mm^3^ with left shift. He underwent computed tomography (CT) of the abdomen and pelvis with intravenous contrast, which demonstrated mesenteric lymphadenitis and mild cecal wall thickening suggestive of possible early colitis. He denied any exposure to COVID-19 or any sick contacts, but because of the ongoing pandemic and high infection rate in our community, he was tested anyway for SARS-CoV-2 by RT-PCR on a nasopharyngeal swab. The test was negative. He was also tested for gonorrhea and chlamydia (GC) by PCR on a urethral swab, the result of which was pending at the time of discharge from the ER. He was managed conservatively and sent home with a plan for close follow-up at a local clinic.

He returned to the ER two days after his initial visit with worsening abdominal pain, persistent fevers, and new pain in his scrotum and left testes. He denied any urinary symptoms or penile discharge. He also denied diarrhea or respiratory symptoms, including cough, sputum production, shortness of breath, or chest pain. The GC PCR from two days prior was positive for *Chlamydia trachomatis* and negative for *Neisseria gonorrhoeae*. His vitals were notable for a temperature of 103.2 F, a pulse of 120 beats/min, a blood pressure of 119/76 mmHg, and an oxygen saturation of 98% on RA. Left-sided epididymal tenderness was noted. Labs were drawn and resulted as shown in Table 1. Blood cultures and retesting for SARS-CoV-2 by PCR on a nasopharyngeal swab yielded negative results. CT of the abdomen and pelvis with intravenous contrast was again suggestive of cecal colitis with surrounding adenopathy at the level of the cecum and terminal ileum. A scrotal ultrasound demonstrated normal appearance and vascularity of both epididymes, normal echotexture of the testes, and symmetric increased blood flow in both testes. The patient was admitted to the medicine service for acute *Chlamydia* epididymitis and started on doxycycline.

**Figure 1:**
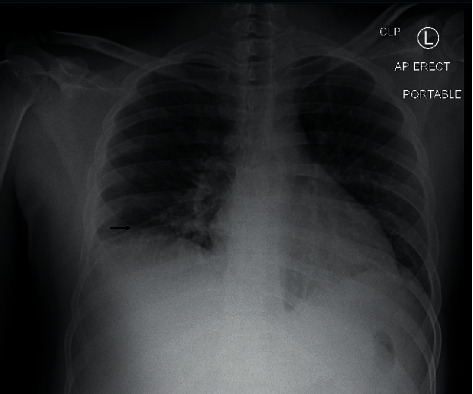
Suboptimal lung inflation with bibasilar atelectasis (identified by arrow).

On the second hospital day, he was noted to be persistently febrile with a temperature >102 F and continued scrotal and testicular pain. His antibiotics were broadened to vancomycin and piperacillin-tazobactam, and doxycycline was continued. A chest X-ray (CXR) done at 24 hours looking for other sources of infection showed bibasilar atelectasis but no consolidation (see Figure 1).

He then began having diarrhea and complained of shortness of breath, with initially normal pulse oximetry. He was started on probiotics and on supplemental oxygen at 2 L/min via nasal cannula for comfort. His breathing continued to deteriorate over the next few hours, and he developed hypoxemia, requiring 6 L O2/min to maintain pulse oximetry >92%. A third RT-PCR for SARS-CoV-2 (over a span of 4 days) was sent, given high suspicion, and it was again negative.

**Figure 2:**
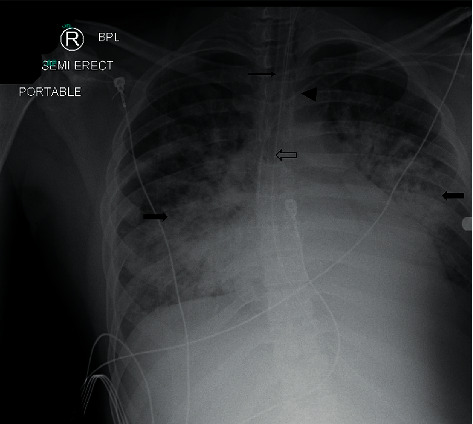
Chest X-ray obtained after endotracheal intubation showing extensive bilateral airspace consolidation (identified by block arrows with color fill) with some sparing of the apices. Tip of the endotracheal tube at the level of the clavicular heads (arrow). Nasogastric tube (block arrow without color fill) and right subclavian line (arrowhead) in place.

At about 36 hours after hospitalization, he developed severe respiratory distress requiring high flow O2 at 15 L/min. CXR now showed bilateral lung infiltrates (see Figure 2). His N-terminal-prohormone brain natriuretic peptide (NT-proBNP) was elevated at 18,953 pg/mL with no prior history of heart failure, and he was started on intravenous diuresis with furosemide. He was transferred to the intensive care unit and started empirically on therapeutic anticoagulation with intravenous heparin for an elevated D-dimer and concerns of venous thromboembolism. CT angiography of the chest to rule out pulmonary embolism could not be performed due to worsening renal function. He underwent endotracheal intubation with initiation of mechanical ventilation 48 hours after hospitalization. His temperature was as high as 105.8 F. He became hypotensive shortly after intubation, requiring pressor support with intravenous norepinephrine.

The nephrology service was consulted for oliguric renal failure with creatinine of 3 mg/dL. His cellular urine sediment consisted of hyaline casts, pyuria, and proteinuria. Initially, the renal failure was presumed to be related to contrast nephropathy, as the patient had undergone two CT scans with intravenous contrast over 48 hours (both ER visits), along with some component of prerenal injury related to hypovolemia. Serological work up including ANA, ANCA, antiphospholipid antibodies, and serum complements was normal.

The patient continued to have persistently high fevers on broad spectrum antibiotics with negative blood, sputum, urine, and genital cultures. He received one dose of ceftriaxone to empirically cover for gonorrhea, despite negative testing. Infectious disease service was consulted. Vancomycin was discontinued after 48 hours of treatment, and piperacillin-tazobactam and doxycycline were continued. A fourth-generation antibody/antigen test for HIV and an IgG antibody screen for syphilis were both negative.

He continued to require high pressor support and high FiO2 on the ventilator, and he remained oliguric with elevated creatinine despite volume resuscitation. An echocardiogram for worsening NT-proBNP>35 K was performed and demonstrated a mildly dilated left ventricle with severe global hypokinesis, a left ventricular ejection fraction (LVEF) of 30%, moderate tricuspid regurgitation, an estimated pulmonary artery systolic pressure of 28–35 mmHg, and a dilated inferior vena cava with an estimated right atrial pressure of 8–15 mmHg. The patient was noted to have pulsus alternans at the time of the echocardiogram suggestive of a low cardiac output state.

The patient's clinical course was not expected for *Chlamydia* epididymitis and led to an increasing suspicion, given the ongoing pandemic, that the patient had an inflammatory syndrome related to prior unrecognized COVID-19, akin to MIS-C, with multiorgan failure involving renal, respiratory, cardiovascular, and gastrointestinal systems. Given the patient's continued clinical deterioration, and while awaiting the result of the antibody testing for SARS-CoV-2, a consensus was reached to treat the patient empirically with a single dose of intravenous tocilizumab 400 mg on day 4 of hospitalization. Within 24 hours of administration of tocilizumab, the patient started to show improvement in his fever. There was also a notable decrease in his FiO2 and norepinephrine requirement. His urine output and renal function started to improve. He was successfully extubated around 48 hours after the administration of tocilizumab, and pressor support was no longer needed. His SARS-CoV-2 immunoglobulin G (IgG) antibody came back positive, confirming prior infection. IgA could not be checked as it was not available locally. He was then started on prednisone 50 mg bid (1 mg/kg/day). Piperacillin-tazobactam was discontinued, and doxycycline was continued for a total of 10 days for treatment of *Chlamydia*.

He was also found to have an acute venous thrombosis in the right upper extremity cephalic vein, and he was switched to oral rivaroxaban. As his blood pressure improved, metoprolol and low-dose lisinopril were added to support his low cardiac output. Oxygen supplementation was discontinued, and a repeat echocardiogram on hospital day 10 showed improvement in the left ventricular ejection fraction to 45–50%. He was discharged home on hospital day 10 with a plan for an additional 2 weeks of prednisone 50 mg twice daily, followed by a slow taper. Metoprolol, lisinopril, and rivaroxaban were continued. NT-proBNP at discharge was 88 pg/mL. Two weeks after discharge, he reported complete resolution of all his symptoms.

## 3. Discussion

COVID-19 can play out in a variety of ways in SARS-CoV-2-infected patients, ranging from no to minimal symptoms to critical illness lasting many weeks and often ending in death [9, 10]. In many patients, there is an initial phase of milder symptoms, lasting about a week, followed by a phase of severe illness, heralded by the onset of rapidly progressive respiratory failure. This biphasic course corresponds temporally with an initial phase of viral replication followed by development of an immune response. However, it is still a matter of debate how much of the pathology of COVID-19 is the result of direct cell damage caused by SARS-CoV-2 and how much is due to the host response [11–15]. That at least part of the observed pathogenesis is immune mediated has been underscored by the discovery that treatment with immunosuppressive drugs such as dexamethasone [16, 17] and possibly tocilizumab [18–21] favorably impacts the course of disease.

The multisystem inflammatory syndrome is thought to represent a hyperinflammatory response triggered by SARS-CoV-2 [22, 23]. 30% of adults with MIS-A and 45% of children with MIS-C reported to CDC had negative RT-PCR for SARS-CoV-2 but positive anti-SARS-CoV-2 serology at the time of diagnosis [8], suggesting that most had cleared the virus before the onset of symptoms. For unknown reasons, there is often an asymptomatic interval lasting up to a few weeks (typically 2–6 weeks) between acute infection and onset of MIS. Thus, the multisystem inflammatory syndrome can be regarded as a postinfectious phenomenon, clinically distinct from the second phase of acute SARS-CoV-2 infection [22, 23].

In this context, we present a case of what we believe to be COVID-19-associated multisystem inflammatory syndrome in an adult patient. He presented with fever, gastrointestinal symptoms, progressive respiratory and renal failure, shock, and heart failure. SARS-CoV-2 RNA was undetectable by RT-PCR from three separate nasopharyngeal swabs obtained over 4 days, but IgG antibody to SARS-CoV-2 was positive. Evidently, he had been previously exposed to SARS-CoV-2, but he did not recall any recent prior exposure or illness. As he had not travelled and as the case occurred 3 months after the arrival of the first wave of cases of COVID-19 in our geographic area in early April 2020, we can be confident that his exposure took place within those 3 months. In contrast to Kawasaki disease, our patient had no physical evidence of vasculitis and no visible coronary aneurysms on echocardiography. He was found to have concomitant *Chlamydia* epididymitis. He improved dramatically after receiving tocilizumab, systemic steroids, and doxycycline. Intravenous immune globulin, which has since emerged as a recommended therapy [23–26], was not administered to this patient.

Bastug et al. [27] and Ahmad et al. [28] described 3 other cases of MIS-A in patients presenting with fever and abdominal symptoms with CT evidence of ileocolic mesentery lymphadenopathy followed by worsening cardiogenic shock, treated with IVIG, steroids, and anakinra. Even in studies [3, 6, 29] published on pediatric patients with MIS-C, gastrointestinal symptoms were noted to be most prominent, like the initial presenting symptom of abdominal pain in our patient.

*Chlamydia trachomatis* is sexually transmitted and has been associated with nongonococcal urethritis and conjunctivitis. It is known to cause cervicitis and pelvic inflammatory disease in women, epididymitis and prostatitis in men, and lymphogranuloma venereum, a disease associated with genital ulcers and painful inguinal lymphadenopathy. *C. trachomatis* has also been associated with a postinfectious syndrome: the triad of reactive arthritis, conjunctivitis or uveitis, and urethritis or cervicitis (Reiter syndrome) [30].

Some unusual manifestations of *Chlamydia* have been reported in the literature. Morgan et al. [31] described a case of severe sepsis and acute myocardial dysfunction in an adolescent with *C. trachomatis* pelvic inflammatory disease in the setting of type 1 diabetes mellitus. There have been case reports of myocarditis of lower severity associated with *C. trachomatis* [32–36]. *Chlamydia pneumoniae* and *Chlamydia psittaci* have also been associated with heart infections [37]. It should be noted that Kawasaki syndrome has been reported in association with a variety of infections, among these, *C. pneumoniae*, though a causal link has never been established [38–42].

Our patient had epididymitis and urethritis attributable to *C. trachomatis* infection. He had none of the autoimmune symptoms of the Reiter syndrome, but had evidence of an acute cardiomyopathy. Although *C. trachomatis* has been associated with myocarditis and myocardial dysfunction in the case reports just described, the scope and severity of illness seen in our patient would be extremely unusual for chlamydial infection. Therefore, we do not believe that chlamydial infection alone could explain the full spectrum of our patient's illness. On the other hand, our patient had evidence of recent prior SARS-CoV-2 infection and exhibited numerous manifestations consistent with multisystem inflammatory syndrome, as described in children and adults. It will be interesting to see if other infections or immune triggers occurring in the weeks following SARS-CoV-2 infection can do the same thing.

## 4. Conclusion

In summary, we have presented a case of an adult patient with acute *Chlamydia trachomatis* infection occurring in the wake of asymptomatic (or at least unrecognized) COVID-19. The intersection of these two infections resulted in a severe illness consistent with COVID-19-associated MIS-A. We suspect that *C. trachomatis* infection in this instance may have set off the multisystem inflammatory syndrome after the immunological stage was set by COVID-19. From a therapeutic standpoint, the excellent response we observed of MIS-A to tocilizumab in conjunction with corticosteroids without the use of intravenous immune globulin is noteworthy. This case also highlights the importance for clinicians who care for adult patients to be familiar with the multisystem inflammatory syndrome, even though it occurs more commonly in the pediatric population.

## Figures and Tables

**Table 1:** Lab values for the patient on the two emergency room (ER) visits and during hospitalization.

	Reference range	1^st^ ER visit	2^nd^ ER visit	72 hours after admission
WBC (10^3^/*µ*l)	4.5–10	11.5	13	23.1
Neutrophils (%)	34–72.6	79.7	92	90.8
Lymphocytes (%)	21.2–53.1	8.1	3.4	2.9
Monocytes (%)	4.7–12.5	11.2	2.8	1.2
Eosinophils (%)	0.8–7.0	0.1	1.0	3.1
Hemoglobin (g/dL)	12.2–16.2	14.2	15.7	12.6
Hematocrit (%)	36–51	41.9	46.8	36.7
Platelet (10^3^/*µ*l)	140–400	178	223	262
Sodium (mmol/L)	136–145	131	129	134
Potassium (mmol/L)	3.5–5.1	3.8	4.0	3.6
Carbon dioxide (mmol/L)	21–32	28	27	20
Blood urea nitrogen (mg/dL)	7–20	9	13	36
Creatinine (mg/dL)	0.44–1.27	1.25	1.77	2.96
Alanine aminotransferase (U/L)	13–61	36	97	71
Aspartate aminotransferase (U/L)	10–37	20	61	72
Alkaline phosphatase (U/L)	45–117	72	119	95
Total bilirubin (mg/dL)	0.2–1.0	0.7	0.9	0.8
Activated partial thromboplastin time (seconds)	25.3–37.0	—	32	43.5 (on iv heparin drip)
Prothrombin time (seconds)	9.4–12.5	—	19	13.1
INR	0.9–1.1	—	1.62	1.14
D-dimer (ng/mL)	0–500	—	3363	2350
Ferritin (ng/mL)	26–388	—	923.9	—
Lactate dehydrogenase (U/L)	87–241	—	265	—
ESR (mm/hr)	0–13	—	99	—
CRP (mg/dL)	0–0.3	—	32	—
NT-proBNP (pg/mL)	0–125	—	18953	>35000

WBC, white blood cell count; INR, international normalized ratio; ESR, erythrocyte sedimentation rate; CRP, C-reactive protein; NT-proBNP, N-terminal-prohormone brain natriuretic peptide.
